# Effects of choline metabolite—trimethylamine N-oxide on immunometabolism in inflammatory bowel disease

**DOI:** 10.3389/fimmu.2025.1591151

**Published:** 2025-07-17

**Authors:** Siyu Wang, Yifan Ni, Shuwei Zhou, Huiping Peng, Ying Cao, Yue Zhu, Jing Gong, Qiulu Lu, Zhongyu Han, Yumeng Lin, Yaodong Wang

**Affiliations:** ^1^ Department of Preventive Medicine, Kunshan Hospital of Chinese Medicine, Kunshan, China; ^2^ Suzhou Key Laboratory of Integrated Traditional Chinese and Western Medicine for Digestive Diseases, Kunshan, China; ^3^ Department of Clinical Nutrition, Kunshan Hospital of Chinese Medicine, Kunshan, China; ^4^ Zhongda Hospital, School of Medicine, Southeast University, Nanjing, China; ^5^ Department of Gastroenterology, Kunshan Hospital of Chinese Medicine, Kunshan, China; ^6^ Department of Proctology, Kunshan Hospital of Chinese Medicine, Kunshan, China; ^7^ Health Management Center, Nanjing Tongren Hospital, School of Medicine, Southeast University, Nanjing, China

**Keywords:** trimethylamine N-oxide, inflammatory bowel disease, gut microbiota, metabolites, immunometabolism

## Abstract

Trimethylamine N-oxide (TMAO), a key metabolite derived from the gut microbial metabolism of choline, has recently been implicated as a significant contributor to the development of several chronic diseases, including diabetes, cardiovascular disease, and chronic kidney disease. Its detrimental effects have garnered widespread attention in the scientific community. Inflammatory bowel disease (IBD), marked by persistent and recurring gastrointestinal inflammation, is a significant global health issue. Emerging evidence highlights a critical role for TMAO in the pathogenesis of IBD. This review comprehensively summarizes current research on the association between TMAO and IBD, with a particular focus on the mechanisms by which TMAO regulates immunometabolism in diseases.

## 1 Introduction

Trimethylamine N-oxide (TMAO) is an endogenous compound predominantly found in marine organisms, with only trace amounts present in most freshwater fish (1). In marine species, TMAO serves essential physiological roles, including osmoregulation, antifreeze activity, and protein stabilization (2, 3). In humans, TMAO is generated from choline- and carnitine-rich foods via gut microbiota activity and subsequent hepatic metabolism (4). Initially regarded as a mere byproduct of choline metabolism with no physiological relevance, TMAO has since been associated with various pathophysiological effects (5–9). This shift in understanding began with the seminal study by Wang et al. in 2011 (10), which demonstrated that dietary choline and TMAO promoted atherosclerosis. In recent years, advancing research has increasingly highlighted the pivotal role of TMAO in human health across multiple domains.

Inflammatory Bowel Disease (IBD) encompasses two primary subtypes: Crohn’s disease (CD) and ulcerative colitis (UC). UC is a chronic and recurrent gastrointestinal disorder driven by immune dysregulation, characterized by alternating episodes of inflammation and remission, and remains incurable (11). CD is a chronic intestinal inflammation that can affect any part of the digestive tract, manifested as segmental and transmural inflammation. Its typical symptoms include abdominal pain, diarrhea, weight loss, and fatigue, and may lead to extraintestinal complications. The prevalence of CD in the population is increasing, and specific environmental factors are associated with its development (12). The etiology of IBD is multifactorial, involving complex interactions among genetic predisposition, environmental factors, the intestinal microbiome, and the immune system (13–16). Recent studies have implicated TMAO, an intestinal microbiota-dependent metabolite, in the pathogenesis and progression of IBD through its role in immune regulation. TMAO has been shown to contribute to IBD by modulating the innate immune response. Notably, elevated TMAO concentrations induce oxidative stress and activate the NOD-like receptor thermal protein domain associated protein 3 (NLRP3) inflammasome in human fetal colon cells, suggesting that TMAO triggers inflammasome activation and promotes an inflammatory response in the intestinal endothelium (17).

Immunometabolism is an emerging research field in which immune cells regulate energy and biosynthesis through pathways such as glycolysis, oxidative phosphorylation, and tricarboxylic acid cycle in different microenvironments to meet proliferation, differentiation, and effector functions (18). Through the “metabolic control” molecular network, immune cells can switch between pro-inflammatory and anti-inflammatory states, and their abnormal regulation is closely related to IBD, rheumatoid arthritis, and tumor immune imbalance (19, 20). Targeted metabolic pathways not only accurately regulate the immune responses, but also provide new strategies for personalized therapy, demonstrating significant clinical application potential.

In this review, we provide an overview of the latest research on TMAO in the context of immunometabolism. We discuss the potential mechanisms by which TMAO affects the immune regulation of IBD and its impact on several immunometabolism-related diseases, aiming to explore strategies for reducing IBD risk through dietary interventions.

## 2 Pathogenesis of IBD

Since its emergence in the western world over a century ago, the epidemiology of IBD has undergone a significant shift (21, 22). The incidence of IBD has risen markedly in developing and newly industrialized countries, while in developed nations, the prevalence continues to increase, particularly among children and older adults. IBD, once considered predominantly a disease of children and young adults, is now increasingly diagnosed in the elderly population (23–25). This growing trend underscores the urgent need to further investigate the pathogenesis of IBD. Although the precise etiology of IBD remains elusive, considerable progress has been made in understanding its underlying mechanisms in recent years. Research suggests that the pathogenesis of IBD is multifactorial, involving genetic predisposition, gut microbiota dysbiosis, environmental factors, and immune system abnormalities (26, 27). In this review, we focus specifically on the roles of intestinal dysbiosis and immune dysfunction in the development of IBD.

### 2.1 Dysbiosis of intestinal flora

A reduction in the abundance and diversity of specific bacterial genera in the gut microbiota, as well as alterations in microbiota-derived metabolites, has been identified as a potential key factor in the pathogenesis of IBD (28, 29). For instance, an analysis of intestinal biopsies and fecal samples from 231 IBD patients and healthy controls, using 16S rRNA gene pyrosequencing, revealed marked variations in microbiota composition between the two groups (30). Morgan et al. discovered that the levels of certain microbiota, such as *Roseburia* and *Phascolarctobacterium*, were markedly decreased in UC and CD patients in contrast to healthy people (30).

*Roseburia* is known for its role in promoting the generation of gut anti-inflammatory regulatory T cells (31), while *Phascolarctobacterium* metabolizes succinate to generate propionate when in co-culture with *Paraprevotella (* 32). Propionate, a short-chain fatty acid (SCFA), has recognized anti-inflammatory properties (33). The reduction in *Phascolarctobacterium* populations in IBD patients may impair the production of propionate, thus diminishing the anti-inflammatory effects of SCFAs and contributing to the exacerbation of IBD symptoms. In contrast, the family *Enterobacteriaceae*, particularly *Escherichia*/*Shigella*, exhibited a significant increase in abundance in the gut microbiota of CD patients. This family has been consistently linked to intestinal inflammation in multiple studies (34–37). Moreover, the abundance of bacteria carrying bile salt hydrolase and bile acid inducible enzyme in the gut microbiota of IBD patients, such as *Firmicutes*, *Ruminococcaceae*, *Lachnospiraceae*, and *Eubacterium*, is reduced. A decrease in these bacterial populations can reduce the metabolic process turning primary bile acids into secondary bile acids (e.g., deoxycholic acid and lithocholic acid) through processes like depolymerization and 7α-dehydroxylation. This alteration leads to an increase in primary and conjugated bile acids (e.g., cholic acid, taurocholate, and glycocholic acid, chenodeoxycholic acid) and a decrease in secondary bile acids. Recent advances in genomics and metabolomics have provided compelling evidence that bile acids and their receptors play a critical role in the pathophysiology of IBD (38–41). As signaling molecules, bile acids can influence the gut microbiota, epithelial barrier integrity, and intestinal immune responses by activating various bile acid receptors, such as the farnesoid X receptor (FXR), vitamin D receptor, and the G protein-coupled bile acid receptor 1 (42).

### 2.2 Immune abnormalities

Immune dysregulation in IBD is characterized by epithelial damage, including abnormal mucus production and defective mucosal repair. This is coupled with an exaggerated inflammatory response driven by the intestinal microbiota, leading to extensive infiltration of various immune cells, including T cells, B cells, dendritic cells (DCs), macrophages, and neutrophils, into the lamina propria. The failure of immunomodulatory mechanisms to regulate this inflammation further exacerbates the condition (43, 44). Activated cells in the lamina propria secrete elevated levels of proinflammatory cytokines, including tumor necrosis factor (TNF), interleukin (IL) -1β, interferon-γ (IFN-γ), and cytokines involved in the IL-23/T helper cell 17 (Th17) signaling pathway (27, 44, 45). Among the various immune mediators, Th17 cells are now recognized as a central pathogenic factor in IBD. For example, a study identified significant infiltration of Th17 cells within the inflamed intestinal mucosa of IBD patients (46). Moreover, the number of cells releasing IL-17 and other Th17-related cytokines was markedly higher in the inflamed tissues of IBD patients compared to normal tissues (46, 47). Additionally, numerous studies have demonstrated that specific gut microbiota play a crucial role in driving the differentiation of Th17 cells (48, 49).

## 3 Microbial-host interplay in TMAO synthesis and metabolism

TMAO originates from dietary precursors metabolized through a tightly coordinated interplay between gut microbiota and host hepatic enzymes (50). This section delineates the biochemical pathways within their biological contexts, emphasizing the roles of specific microbial taxa and host factors.

### 3.1 Gut microbiota: primary producers of trimethylamine

Gut microbiota, particularly *Firmicutes* and *Proteobacteria*, are key mediators of TMA production through distinct enzymatic pathways (51, 52). In choline metabolism, *Firmicutes*, *Actinobacteria*, and *Proteobacteria* utilize the CutC/D enzyme system to cleave choline—abundant in eggs, meat, and fish (53)—into TMA, a function absent in *Bacteroidetes (* 54). L-carnitine, derived from red meat and dairy (55), is directly converted to TMA by *Proteobacteria* and some *Firmicutes* via the CntA/B system (56, 57). Alternatively, it can be metabolized into γ-butyrobetaine or betaine (58, 59), with γ-butyrobetaine further transformed into TMA by YeaW/X (60), enzymes specific to *Gammaproteobacteria (* 61). Betaine, sourced from cereals and leafy vegetables (53, 62), yields TMA via low-efficiency pathways involving betaine reductase or betaine-homocysteine-mediated demethylation (59, 63). Additionally, ergothioneine—present in mushrooms and organ meats (64)—is degraded by *Burkholderia* through ergothionease to generate TMA (65). Notably, ergothioneine’s anti-inflammatory and neuroprotective roles contrast with the pro-atherogenic effects of TMAO, underscoring the need for deeper mechanistic insights (66).

Different gut microbes vary in their ability to generate TMA. Metagenomic and biochemical surveys have identified key bacterial taxa and genes involved in TMA synthesis. In particular, many *Firmicutes* carry the cutC/D genes and convert choline or carnitine to TMA. For example, *Clostridium* sp*orogenes*, *C. hathewayi*, *C. asparagiforme* and related Clostridia are known TMA producers (67, 68). Other Firmicutes *genera* such as *Anaerococcus*, *Roseburia* and *Ruminococcus* have also been linked to TMA formation (67). Some Proteobacteria harbor the Rieske-type cntA/B enzymes that convert L-carnitine into TMA, and certain Actinobacteria and Bacteroidetes also carry cut-like genes. In aggregate, recent human studies find that gut communities rich in these taxa tend to have higher TMAO output. For example, a large cohort study identified eight Firmicutes species, one *Bacteroides* genus, and one *Actinobacterium* whose abundances correlate strongly with plasma TMAO levels (67). *In vitro* and metagenomic screens also confirm that the cutC gene is concentrated in Clostridia and related anaerobes, while cntA is found chiefly in Gammaproteobacteria. Thus the composition of the gut microbiome and the balance between TMA-producing taxa and others critically influence how much TMA is made from a given diet (69).

### 3.2 Host hepatic oxidation: from TMA to TMAO

Following intestinal absorption, TMA is rapidly oxidized in the liver by flavin monooxygenases (FMOs), specifically FMO1 and FMO3. FMO3 is the dominant hepatic isoform in adults, exhibiting 10-fold higher activity than fetal-expressed FMO1 (70). FMO3 efficiently converts absorbed TMA into TMAO, regardless of its precursor origin. The efficiency of TMAO production varies depending on the precursor compounds. Among these, choline is the most potent precursor, producing TMAO more efficiently than either betaine or L-carnitine. Additionally, L-carnitine produces TMAO at the fastest rate, while betaine generates TMAO more slowly (71).

### 3.3 Systemic fate and excretion

**Figure 1:**
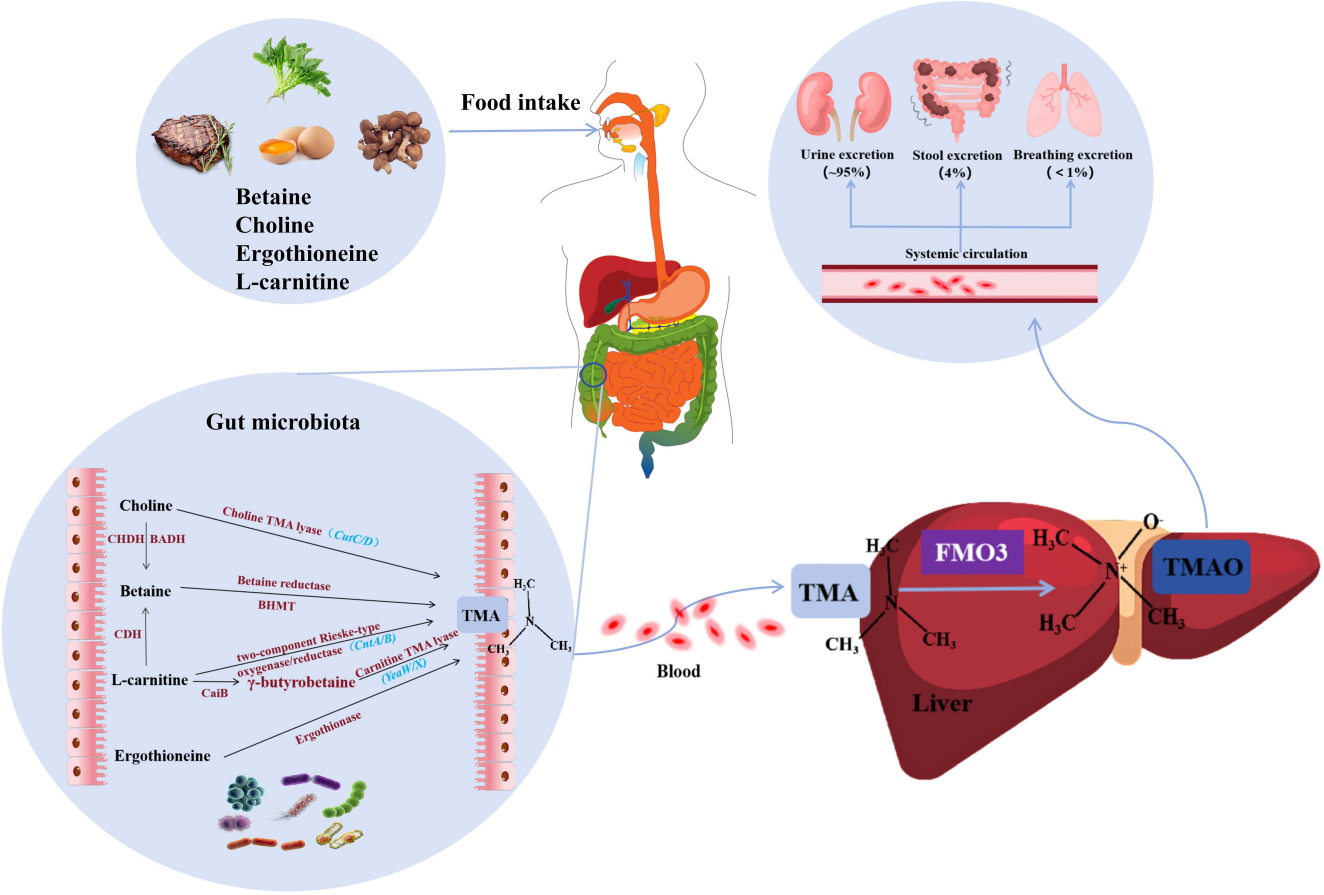
Synthesis and metabolism of TMAO. Precursors of TMA (including L-carnitine, choline, ergothionein) can be ingested from foods such as red meat, eggs, and mushrooms, and then they are converted to TMA by various enzymes under the action of gut microbiota. TMA is absorbed into the bloodstream in the intestine and rapidly oxidized by FMO3 in the liver to form TMAO. Finally, transported through the systemic circulation to other organs, about 95% of TMAO is excreted in the urine, only 4% in the feces, and less than 1% in the breath. TMA, trimethylamine; FMO3, flavin-containing monooxygenase 3; TMAO, Trimethylamine oxide; CHDH, Choline dehydrogenase; BADH, Betaine-aldehyde dehydrogenase; BHMT, Betaine-homocysteine methyltransferase; CDH, L-carnitine dehydrogenase; CaiB, γ-butyrobetainyl-CoA:carnitine CoA transferase.

Once produced, most TMAO enters the systemic circulation, with a portion being taken up by extrahepatic tissues. However, the majority of TMAO is excreted unchanged in the urine within 24 hours, with only a small amount excreted in the feces (72) (**Figure 1**).

## 4 Dual roles of TMAO in immunometabolism

The field of immunometabolism has made significant strides over the past decade, highlighting the critical role of intracellular metabolism in regulating immune cell function. Although the mechanisms by which metabolites influence the immune system are complex, they can generally be categorized into two main types. The first category involves metabolites serving as essential nutrients, energy sources, and building blocks required for the development, differentiation, proliferation, and functional activity of immune cells. The second category encompasses metabolites that function as signaling molecules, which immune cells detect via receptors expressed on various subcellular organelles, including the cell membrane, cytoplasm, mitochondria, and nucleus. These two mechanisms are now widely recognized as integral to the immune response, with cell-specific and systemic metabolism being tightly interlinked. The proper functioning of both immune cells and metabolism is mutually dependent. Consequently, the immunometabolism interface—which encompasses both local metabolism within immune cells and the broader systemic metabolism of the immune system—has emerged as a promising therapeutic target for various chronic diseases (73, 74).

Given the importance of the immunometabolism interface as a therapeutic target for chronic diseases, it is essential to explore the impact of specific metabolites on different types of immune cells. One such metabolite that has attracted considerable interest is TMAO. Understanding how TMAO affects various immune cells, including macrophages, DCs, T lymphocytes, B lymphocytes, and natural killer (NK) cells, is of great significance in further unraveling the complex mechanisms of immunometabolism and potentially developing new therapeutic strategies.

### 4.1 Impact of TMAO on macrophages

Macrophages, as pivotal components of the innate immune system, play crucial roles in mediating inflammatory responses (75). TMAO has been demonstrated to influence macrophage polarization and activation, thereby exacerbating inflammatory conditions (76). Studies reveal that TMAO promotes macrophage polarization towards the M1 phenotype, characterized by enhanced production of pro-inflammatory cytokines and amplified antigen-presenting capacity. This polarization is critical for developing Th1 and Th17 responses that accelerate the progression of inflammatory disorders such as graft-versus-host disease (77). In TMAO-treated macrophages, significant upregulation is observed in both M1 signature cytokines (including IL-1β, IL-6, TNF-α, Chemokine (C-X-C motif) ligand (CXCL) 9, and CXCL10) and key genes such as NLRP3 (77).

The NLRP3 inflammasome, a critical protein complex in macrophage activation, is directly activated by TMAO through caspase-1 cleavage and subsequent IL-1β secretion - pivotal mediators of inflammatory cascades. Mechanistically, TMAO stimulates nuclear factor kappa-B (NF-κB) nuclear translocation, thereby enhancing transcriptional upregulation of NLRP3 and other pro-inflammatory genes. This process constitutes an essential priming signal for NLRP3 inflammasome activation. Furthermore, TMAO induces reactive oxygen species (ROS) generation in macrophages, creating a feed-forward loop that amplifies both inflammatory responses and oxidative stress (10).

### 4.2 Impact of TMAO on DCs

DCs, as the most potent professional antigen-presenting cells in the body, play a pivotal role in antigen recognition and signal transmission during immune response. The biological characteristics of DCs can be divided into two key stages: immature DCs possess remarkable tissue migration ability and antigen capture efficiency, while mature DCs, through high expression of antigen-presenting molecules and co-stimulatory molecules, specifically activate naive T lymphocytes, thereby playing a core hub role in the initiation, regulation, and maintenance of the immune response (78).

In recent years, studies have shown that TMAO can regulate the immune function of DCs through multiple pathways. Firstly, at the molecular expression level, TMAO can significantly up-regulate the expression of MHC-II molecules and co-stimulatory molecules CD86 on the surface of DCs. By enhancing the stability of antigen peptide-MHC complexes and the intensity of co-stimulatory signals, it effectively improves the capacity of DCs to activate T cells (79). Secondly, in terms of cytokine network regulation, TMAO can promote the secretion of pro-inflammatory factors such as IL-6 and IL-12p40 by DCs, while inhibiting the production of anti-inflammatory factor IL-10. This bidirectional regulatory effect disrupts immune homeostasis and promotes the cascade amplification of inflammatory responses. It is worth noting that TMAO can also significantly increase the level of ROS in DCs through the mitochondrial dysfunction pathway. When the accumulation of ROS exceeds the physiological threshold, it can induce cell apoptosis by activating the caspase pathway, ultimately leading to a reduction in the number of DCs and immune dysfunction (80).

### 4.3 Impact of TMAO on T lymphocytes

TMAO can enhance the activation of effector T cells, shown in increased percentages of IFN-γ^+^ TNF-α^+^ CD8^+^ and CD4^+^ T cells, and upregulated CD44 expression on CD8^+^ and CD4^+^ T cells. After TMAO treatment, in tumor-associated macrophages, stimulatory markers like MHC-I, MHC-II, and CD86 are upregulated, while the anti-inflammatory marker Arg1 is downregulated (79). This indicates a shift of tumor-associated macrophages towards an immune-stimulatory phenotype, which can enhance T cell activation.

Also, TMAO can modulate macrophage phenotype to support T cell responses, promoting effector T cell proliferation and survival (77). Experimental data shows that macrophages treated with TMAO can significantly increase the expression levels of IFN-γ, Ki-67, CD103, and CD44 in CD8^+^ and CD4^+^ T cells, indicating enhanced T cell activation and proliferation. Moreover, TMAO can affect T cell transcriptional profiles. Activated functions include CD8^+^ T lymphocyte proliferation, cellular immune responses, antigen expression, and cell death in tumor cell lines. Inhibited functions involve regulatory T lymphocyte numbers, tumor cell proliferation, and proliferation of fibrous tissue tumors and bone marrow cells (79).

### 4.4 Impact of TMAO on B lymphocytes

Although there’s little direct research on TMAO’s specific impact on B lymphocytes, existing evidence suggests TMAO may indirectly affect B cell function by influencing immune responses. TMAO can combine with and trigger the ER stress kinase PERK, specifically stimulating the PERK arm of the unfolded protein response (UPR). This activation process activates Forkhead box protein O1 (FoxO1), which is a significant contributor to metabolic diseases. Since B lymphocytes are secretory cells highly dependent on ER function, TMAO’s regulation of UPR may impact B cell development, differentiation, or antibody production (81). Regarding inflammatory responses, TMAO reportedly promotes vascular inflammation by activating the NF-κB signaling pathway, increasing endothelial adhesion molecule expression and white blood cell recruitment (82). While this study mainly focuses on endothelial cells, NF-κB is also crucial for B cell activation and function. Thus, TMAO-induced NF-κB activation may influence B lymphocyte responses.

In summary, despite limited direct evidence on TMAO’s impact on B lymphocytes, its ability to regulate pathways like UPR and NF-κB, which are essential for B cell function, suggests potential indirect effects. Further studies are required to clarify the specific mechanisms by which TMAO impacts B cell biology.

### 4.5 Impact of TMAO on NK cells

NK cells are important immune cells in the body, which are not only involved in anti-tumor, antiviral infections, and immune regulation, but also participate in the occurrence of autoimmune diseases in some cases, and can recognize target cells and killing mediators (83). TMAO has been shown to affect the cytotoxicity of NK cells. Elevated levels of TMAO may impair the ability of NK cells to lyse target cells, thereby potentially impairing immune surveillance against tumors and infections (84). Meanwhile, TMAO can alter the production of cytokines by NK cells. Changes in cytokine secretion profiles may affect the regulation of immune responses, inflammation, and immune cell communication (85). In order to comprehensively understand the effects of TMAO on NK cells, further research is necessary to elucidate these mechanisms and their impact on immune related diseases.

## 5 TMAO-mediated immunometabolism in IBD

**Figure 2:**
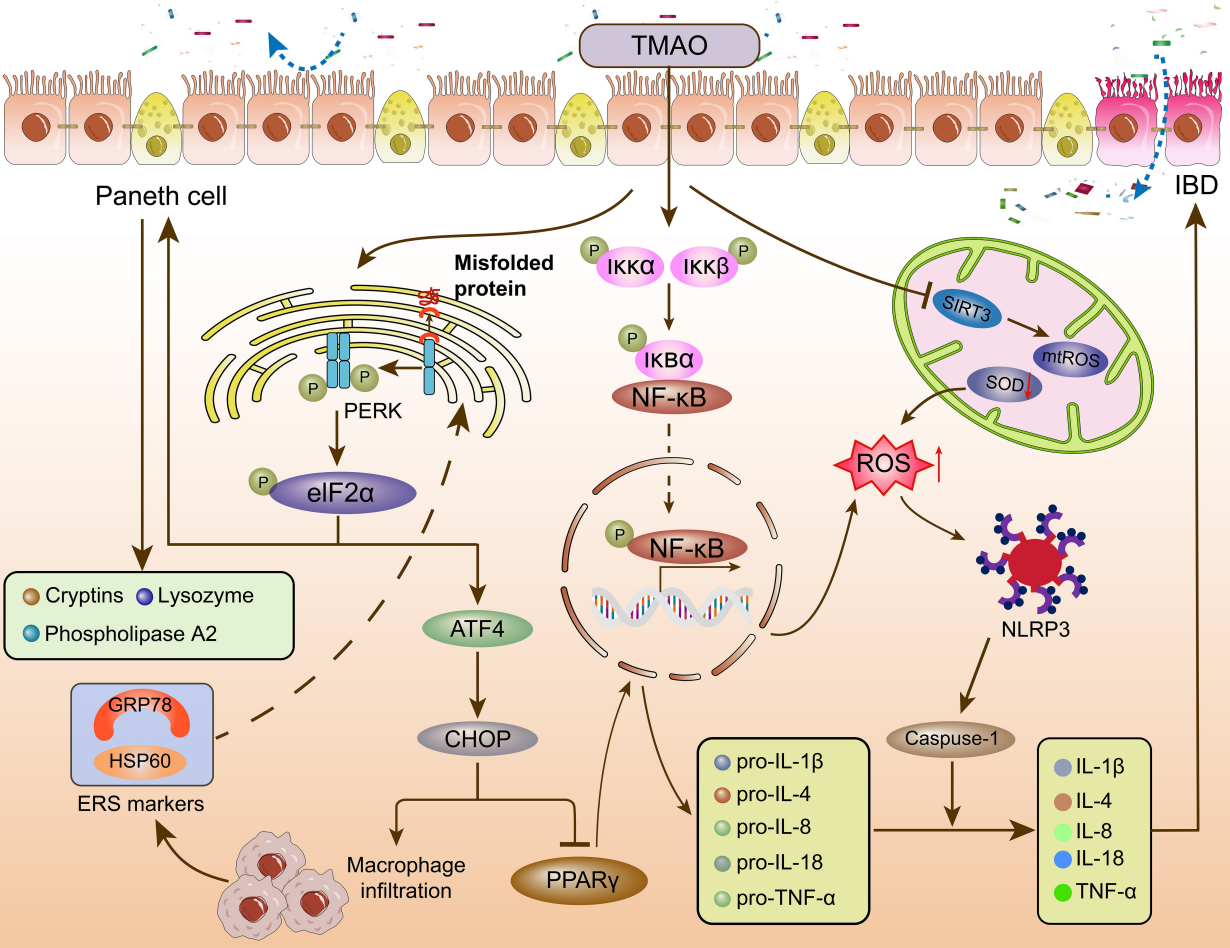
The effect of TMAO on the immunometabolism of IBD. TMAO affects intestinal homeostasis and immune response through PERK, NF-κB, and NLRP3 signaling pathways. TMAO, Trimethylamine oxide; PERK, protein kinase R-like ER kinase; eIF2α, eukaryotic initiation factor 2α; ATF4, activating transcription factor 4; CHOP, C/EBP homologous protein; PPARγ, peroxisome proliferator-activated receptor γ; HSP60: Heat shock protein 60; GRP78: glucose regulated protein 78kD; Iκκ, inhibitor of kappa B kinase; IκB, inhibitor of NF-κB; NF-κB, nuclear factor kappa-B; SIRT3, NAD-dependent protein deacetylase sirtuin-3; ROS, reactive oxygen species; mtROS, mitochondrial reactive oxygen species; SOD, Superoxide dismutase; NLRP3, nucleotide-binding oligomerization domain-like receptor protein 3; Caspase-1, cysteinyl aspartate specific proteinase 1; (IL)-1β, 4, 8, 18, (Interleukins) -1β, 4, 8, 18; TNF-α, tumor necrosis factor-α; IBD, inflammatory bowel disease.

Emerging evidence highlights TMAO’s dual role as both a biomarker of microbial-host metabolic crosstalk and a direct instigator of immune-inflammatory cascades, bridging gut dysbiosis to intestinal mucosal damage (86). Mechanistically, TMAO exacerbates IBD pathogenesis by modulating key signaling pathways—including endoplasmic reticulum stress (ERS) via protein kinase RNA-like endoplasmic reticulum kinase (PERK) activation, NF-κB-driven proinflammatory cytokine production, and NLRP3 inflammasome-mediated pyroptosis—thereby disrupting intestinal epithelial integrity, amplifying macrophage and T cell activation, and skewing immune homeostasis toward a proinflammatory milieu. Notably, recent studies have elucidated how TMAO’s tissue-specific interactions with these pathways converge to perpetuate oxidative stress, barrier dysfunction, and unresolved inflammation, hallmarks of CD and UC. The following sections dissect the molecular interplay between TMAO, PERK, NF-κB, and NLRP3, providing a roadmap to understand how microbial metabolites shape immune outcomes in IBD (**Figure 2**).

### 5.1 TMAO and endoplasmic reticulum kinase PERK

TMAO, a gut microbiota-derived metabolite, has emerged as a critical modulator of ERS through its interaction with the PERK pathway. A landmark study by Chen et al. identified PERK as a specific receptor for TMAO, demonstrating that TMAO directly activates the PERK branch of the UPR at physiological concentrations, thereby inducing the transcription factor FoxO1, which is implicated in metabolic and inflammatory diseases (81). Recent advancements further highlight TMAO’s role in activating PERK across diverse tissues. For instance, Yang et al. observed that chronic TMAO exposure in zebrafish models induced hepatic lipid accumulation, inflammatory infiltration, and fibrosis, accompanied by PERK pathway activation in liver tissues and *in vitro* cell systems (87). These findings underscore PERK as a central mediator of TMAO-driven ERS and inflammation. Notably, TMAO also amplifies ERS markers such as heat shock protein 60 and glucose regulated protein 78kD in macrophages, correlating with elevated toll-like receptor 4 expression and proinflammatory cytokine production (e.g., TNF-α, IL-6) (88, 89).

In IBD, the PERK-C/EBP homologous protein (CHOP) axis plays a key role in linking ERS to mucosal inflammation (81). During sustained ERS, PERK phosphorylates eukaryotic initiation factor 2α (eIF2α), leading to activating transcription factor 4 (ATF4)-mediated upregulation of the pro-apoptotic transcription factor CHOP (90, 91). CHOP is markedly elevated in the intestinal epithelium of IBD patients and murine models, where it exacerbates colitis by inhibiting peroxisome proliferator-activated receptor γ (PPARγ), a negative regulator of NF-κB. This inhibition facilitates NF-κB nuclear translocation, driving the expression of proinflammatory cytokines (e.g., IL-8) and recruiting macrophages to inflamed tissues (92).

In addition to the CHOP signaling pathway, eIF2α, a direct substrate of the PERK pathway, has also been implicated in immune responses in IBD (93). Cao et al. (94) reported that phosphorylation of eIF2α plays a critical role in maintaining the function of intestinal paneth cells and mucosal homeostasis. This is achieved by activating the UPR signaling and promoting the recruitment of messenger RNA to ER membranes for translation. Pyramid-shaped columnar epithelial cells and paneth cells, located at the base of small intestinal crypts (95), are crucial for innate immunity and host defense against fungi, bacteria, and certain viruses. They accomplish this by secreting antimicrobial factors, such as lysozyme, cryptins (alpha-defensins), and phospholipase A2 (96). Defective protein secretion within Paneth cells has been linked to Crohn’s ileitis. Meanwhile, for colonic goblet cells, which are another type of secretory cell, their impaired differentiation and function have been associated with UC (97–99). Additionally, double-stranded RNA-activated protein kinase (PKR) has been identified as a key transducer of inflammatory signals in colonic epithelial cells. PKR promotes intestinal epithelial cell homeostasis and survival by activating the eIF2α-mediated UPR, as well as the STAT3 and AKT signaling pathways. In the absence of PKR, intestinal epithelial cells show impaired survival and proliferation, which exacerbates intestinal inflammation (100). In experimental colitis models, ERS inhibitors such as sodium phenylbutyrate can significantly improve intestinal inflammation, indicating the important role of the ER stress PERK axis in IBD (101). Although there is a lack of specialized research on the direct impact of TMAO on IBD through the PERK pathway, considering TMAO’s ERS inducing ability and the critical role of the PERK pathway in IBD, the relationship between the two deserves further in-depth investigation.

In conclusion, TMAO likely modulates immune responses in IBD through the activation of the PERK-CHOP and PERK-eIF2α pathways, underscoring the complex interplay of UPR signaling in maintaining intestinal homeostasis and immune function.

### 5.2 TMAO and NF-κB signaling

NF-κB is a master regulator of inflammation, and its activation by TMAO has been extensively documented. Seldin et al. demonstrated that TMAO activates NF-κB in aortic endothelial cells via mitogen-activated protein kinase (MAPK)/extracellular regulated protein kinases (ERK) signaling, promoting leukocyte adhesion and vascular inflammation (82). Through pharmacological inhibition, the study further confirmed that NF-κB activation was essential for TMAO-induced expression of inflammatory genes and the subsequent endothelial cell adhesion of leukocytes. These findings align with recent advancements in understanding NF-κB’s role in IBD, where it orchestrates both innate and adaptive immune responses.

TMAO’s proinflammatory effects in IBD are mediated through NF-κB’s dual role in epithelial barrier dysfunction and immune cell activation. In colonic epithelial cells, TMAO induces NF-κB-dependent upregulation of IL-8 and TNF-α, disrupting tight junctions and increasing permeability (17). A study revealed that TMAO synergizes with gut-derived lipopolysaccharides (LPS) to amplify NF-κB activation in dendritic cells, enhancing Th17 differentiation and IL-17 production, which are critical drivers of mucosal damage in UC (102, 103). Furthermore, TMAO-induced NF-κB activation suppresses regulatory T cell (Treg) function by downregulating forkhead box protein P3 expression, thereby tipping the balance toward proinflammatory Th1/Th17 dominance (104–106).

Recent clinical data also implicate dietary TMAO precursors in NF-κB activation. Several studies found that high red meat consumption (a major TMAO source) increased colonic NF-κB activity by 2.3-fold in UC patients compared to plant-based diets (107, 108). These findings underscore the importance of dietary interventions in modulating TMAO-driven inflammation. In addition, studies have found that TMAO increases NADPH oxidase activity, induces the production of ROS, and an increase in ROS can promote the translocation of NF-κB from the cytoplasm to the nucleus (68). Moreover, TMAO can also affect the phosphorylation cascade. A molecular medicine report suggests that TMAO promotes p65 NF-κB subunit phosphorylation when stimulating macrophages, while hydrogen sulfide inhibits this process by upregulating silent information regulator 1 (SIRT1) (a negative regulator of NF-κB) (109). In other words, TMAO may indirectly enhance NF-κB signaling by inhibiting SIRT1. Research has found that NF-κB activation can lead to the amplification of intestinal inflammation and exacerbate mucosal barrier damage. NF-κB not only induces canonical pro-inflammatory cytokines (TNF-α, IL-6, pro-IL-1β) but simultaneously up-regulates facilitative glucose transporter, metabolically locking macrophages, DCs and activated T cells into a glycolytic, NO-rich phenotype that fuels chronic mucosal inflammation (11, 110–112). Therefore, TMAO induced activation of the NF-κB signaling pathway may be an important cause of IBD pathogenesis.

### 5.3 TMAO and the inflammasome NLRP3

The activation of the NLRP3 inflammasome is a critical mediator of TMAO-induced pro-inflammatory effects. In colonic epithelial cells, TMAO has been shown to trigger inflammasome activation and ROS production in a dose- and time-dependent manner. Notably, NLRP3 activation is strongly associated with IBD (113). TMAO activates the NLRP3 inflammasome through three distinct signaling mechanisms: mitochondrial dysfunction induced by Rac1-NOX2-dependent ROS production, which causes cytosolic release of oxidized mtDNA; uptake of nanoscale TMAO aggregates that triggers lysosomal rupture and subsequent cathepsin B release; and ROS-mediated dissociation of Thioredoxin-interacting protein (TXNIP) from thioredoxin, thereby enabling TXNIP-NLRP3 interaction. These signals collectively trigger assembly of the NLRP3-ASC-caspase-1 complex, resulting in mature IL-1β/IL-18 secretion and gasdermin-D-mediated pyroptosis (101). This pyroptotic cascade further suppresses oxidative phosphorylation and sustains glycolytic metabolism (114). Furthermore, TMAO has been found to enhance macrophage infiltration, promote M1 polarization, and stimulate Th1 and Th17 differentiation in allogeneic graft-versus-host disease (77). Interestingly, inhibition of NLRP3 inflammasome activation reversed the M1 polarization of TMAO-stimulated macrophages, suggesting that NLRP3 plays a crucial role as a proteolytic activator in TMAO-induced macrophage responses.

Yue et al. (17) treated fetal human colon cells with TMAO for 3–24h to study colonic inflammation. Autophagy markers (ATG16L1) and NLRP3 inflammasome components were analyzed by Western blotting, qRT-PCR, and immunofluorescence. Adenoviral vectors were used for ATG16L1 overexpression, and siRNA for NLRP3 knockdown. Results showed TMAO inhibited autophagy and activated NLRP3 inflammasome, which were reversed by ATG16L1 or NLRP3 knockdown, indicating a mechanistic link in IBD pathogenesis. The NLRP3 inflammasome is significantly upregulated in the colonic mucosa of patients with UC, and its activity correlates with disease progression (115). Genetic studies on CD have also linked mutations in NLRP3-associated single nucleotide polymorphisms to increased susceptibility to CD (116). Clinically, NLRP3 polymorphisms are linked to increased CD susceptibility, and its activity correlates with UC severity (117). Emerging therapies, such as NLRP3 inhibitors and nanoligomers, show promise in attenuating TMAO-driven inflammation (118). These findings position NLRP3 as a therapeutic nexus for TMAO-mediated IBD.

The interplay between TMAO, PERK, NF-κB, and NLRP3 underscores a multifactorial pathogenesis in IBD. Contrary to predictions based on experimental models, clinical studies have consistently reported reduced plasma TMAO levels in patients with IBD. In a pivotal study, Wilson et al. demonstrated that individuals with IBD had significantly lower plasma TMAO concentrations compared to healthy controls Notably, patients with UC exhibited particularly low levels, markedly less than those observed in individuals with inactive disease (119, 120). These findings have been independently replicated in subsequent research, including a recent study by Laryushina et al., which confirmed that TMAO levels in UC patients vary according to disease activity (121, 122). Furthermore, a Mendelian randomization analysis by Banno et al. supported an inverse relationship between plasma TMAO levels and IBD risk, suggesting that TMAO may exert a protective, rather than pathogenic, role in the context of IBD (123). The reduced TMAO levels observed in IBD likely reflect gut microbiota dysbiosis and altered host metabolism. Disruption of TMA-producing bacteria, reduced availability of dietary precursors, impaired hepatic FMO3 activity, and increased renal clearance under inflammatory conditions all contribute to diminished TMAO synthesis (124–128). These findings suggest TMAO is more indicative of microbial disruption than a driver of IBD pathogenesis, highlighting limitations in translating experimental models to clinical disease (129). In conclusion, TMAO orchestrates immune dysregulation in IBD through PERK-mediated ERS, NF-κB activation, and NLRP3 inflammasome signaling. Future research should prioritize clinical trials to validate these mechanisms and explore personalized therapies targeting the gut-microbiota-TMAO axis.

## 6 TMAO and other diseases

**Figure 3:**
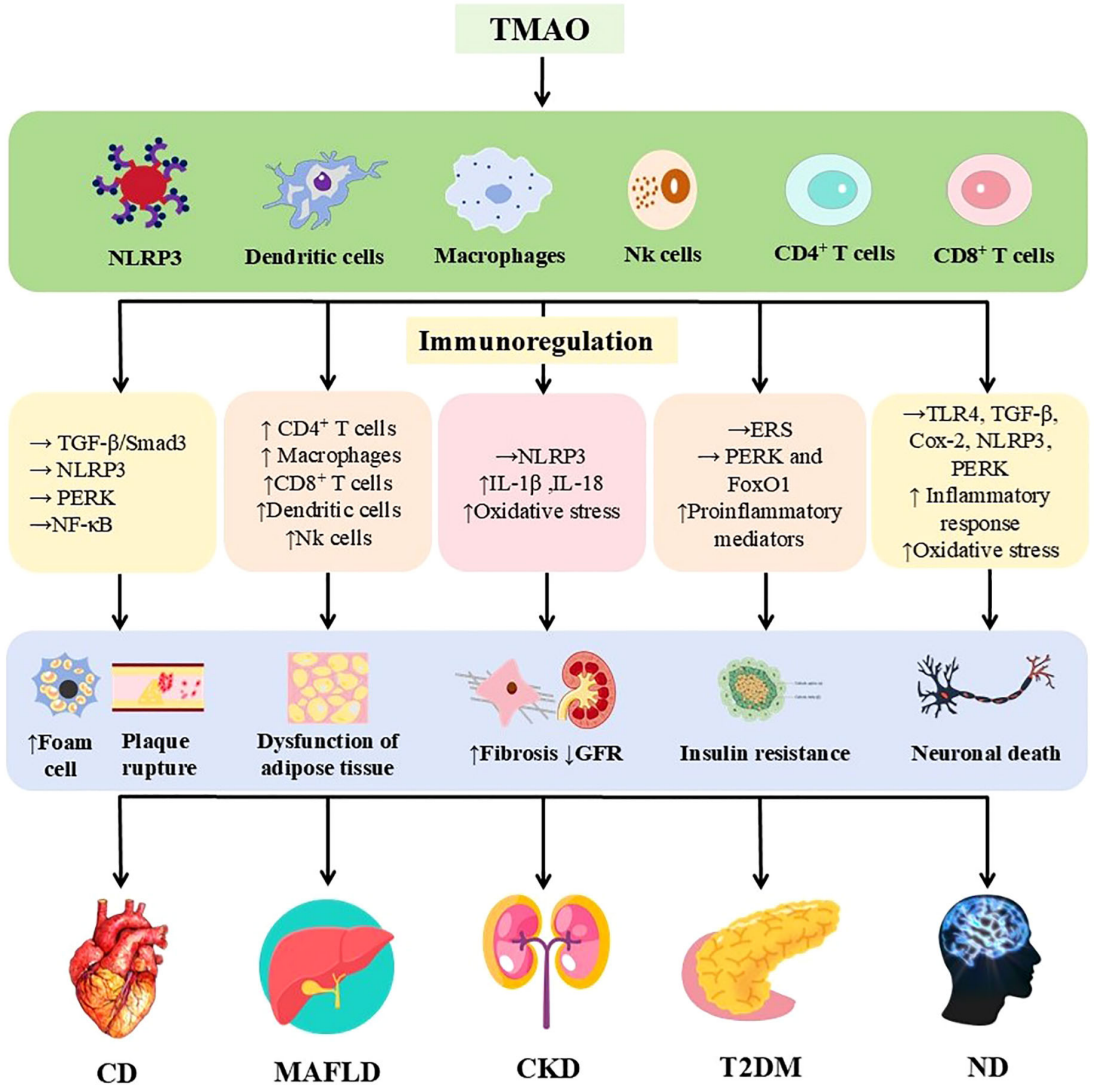
Immunomodulatory effects of TMAO in a variety of chronic diseases. TMAO is involved in cardiovascular disease, MAFLD, chronic kidney disease, type 2 diabetes mellitus, and nervous system diseases by triggering inflammatory responses and regulating the immune system through various mechanisms. The upper arrow indicates increased, the lower arrow indicates decreased, and the horizontal arrow indicates activated. TMAO, Trimethylamine oxide; NLRP3, nucleotide-binding oligomerization domain-like receptor protein 3; TGF-β, transforming growth factor-β; Smad3, Mothers against decapentaplegic homolog 3; NLRP3, nucleotide-binding oligomerization domain-like receptor protein 3; (IL)-1β, 18, (Interleukins) -1β, 18; ERS, endoplasmic reticulum stress; FoxO1, Forkhead box protein O1; TLR4, Toll-like receptor 4; Cox-2, Cyclooxygenase-2; GFR, glomerular filtration rate; MAFLD, metabolic associated fatty liver disease.

Beyond IBD, TMAO’s role in immunometabolism and its involvement in diverse chronic diseases is increasingly recognized (**Figure 3**).

### 6.1 TMAO and cardiovascular disease

Elevated circulating TMAO is strongly linked to cardiovascular and cerebrovascular diseases. Observational studies in Caucasian populations associate doubled TMAO levels with higher mortality, sudden cardiac death, first cardiovascular events, and death risk (130). Prospective cohort studies have shown that long-term elevated plasma TMAO levels significantly increase the risk of cardiovascular disease. The group of healthy women with the highest TMAO increase within 10 years had a 58% increased risk of coronary heart disease (RR 1.58), while those with sustained high TMAO had the highest risk (RR 1.79) (131); The risk of first onset atherosclerotic cardiovascular disease (ASCVD) in the highest baseline TMAO group of the elderly in the community increased by 21% (HR 1.21), and the risk of recurrence in ASCVD patients increased by 25% (HR 1.25), of which the association was stronger in patients with renal insufficiency (132). Mechanistically, TMAO contributes to endothelial dysfunction, an early atherosclerosis feature characterized by increased ROS and pro-inflammatory factors (133). It promotes inflammation via NF-κB pathway activation (82) and by activating the NLRP3 inflammasome in endothelial cells and arterial walls in mice, involving mitochondrial ROS, TXNIP, and lysosomal instability (101, 134).

TMAO accelerates atherosclerosis through multiple mechanisms. In ApoE^-/-^ mice on a high-fat diet (HFD), TMAO supplementation significantly increased vascular plaque progression, macrophage recruitment, and CD36/pro-inflammatory cytokine expression. *In vitro*, inhibiting MAPK/c-Jun n-terminal kinase (JNK) reduced TMAO-induced CD36 expression and foam cell formation (135). Furthermore, TMAO increased aortic plaque area and altered bile acid composition in male ApoE^(-/-)^ mice, accelerating aortic lesions by modifying bile acid profiles, activating FXR and small heterodimer partner, and inhibiting bile acid synthesis (136).

### 6.2 TMAO and chronic kidney disease

As a small organic amine oxide, ~95% of ingested TMAO is renally excreted (50, 76). TMAO serves as a prognostic marker for survival in CKD patients, correlating with glomerular filtration rate, C-reactive protein, and cystatin C (137). In a 5-year study of 521 stable CKD patients, plasma TMAO levels increased over time, and long-term dietary TMAO intake directly associated with renal fibrosis and dysfunction progression (137). Animal models confirm TMAO’s pathogenic role: reducing plasma TMAO slows CKD progression (138), while TMAO accelerates diabetic nephropathy by activating NLRP3 inflammasome, promoting IL-1β/IL-18 release, and worsening renal dysfunction/fibrosis (139).

### 6.3 TMAO and neurological disorders

Detection of TMAO in human cerebrospinal fluid suggests a link to neurodegenerative diseases (140). TMAO is identified as a metabolite significantly associated with Alzheimer’s disease (AD) aspects and shares genetic pathways with AD biomarkers (141). High TMAO levels correlate with early neurological deterioration in acute ischemic stroke patients, potentially mediated by inflammatory markers like IL-6 and C-reactive protein (142).

Elevated TMAO downregulates hippocampal antioxidant enzyme MsrA, contributing to neuroinflammation and cognitive decline post-surgery in aged rats (143). High circulating TMAO correlates with increased brain proinflammatory cytokines and astrocyte activation markers (144). TMAO-mediated neurodegeneration involves activation of peripheral/central inflammatory pathways (NF-κB, NOD-, LRR-, NLRP3 inflammasomes, MAPK/JNK) (145). TMAO levels rise with age-related cognitive dysfunction, inducing mitochondrial dysfunction, oxidative stress, neuronal senescence, and synaptic damage.

### 6.4 TMAO and type 2 diabetes

Clinical studies consistently link higher serum TMAO to T2DM compared to prediabetes or controls (146). Acarbose treatment significantly reduces TMAO levels and improves insulin resistance in T2DM patients (147). T2DM and T2DM-CKD patients exhibit increased gut microbiota TMA-producing bacteria and significantly higher serum TMAO, positively correlating with zonulin, LPS, inflammation, and endothelial dysfunction biomarkers (148). A meta-analysis (12 studies, n=15,314) found high circulating TMAO associated with increased diabetes risk (OR=1.89); each 5 µmol/L plasma TMAO increase raised DM incidence OR by 54% (OR=1.54), showing a positive dose-dependent relationship (149).

Animal studies confirm TMAO’s impact: serum TMAO increase correlated with impaired glucose tolerance in monkeys on HFD (150). TMAO-dependent N-nitros compounds may drive insulin resistance and diabetes. TMAO supplementation reduces bile acid pools, inducing inflammation and T2DM (149). TMAO induces ERS via PERK/FoxO1 activation, decreasing insulin receptor expression and causing insulin resistance (81). Insulin normally suppresses liver FMO3 expression via phosphoinositide 3-kinase; insulin resistance abrogates this suppression, increasing FMO3 expression and plasma TMAO, positioning TMAO as a liver insulin resistance marker (151).

### 6.5 TMAO and metabolic associated fatty liver disease

MAFLD, closely linked to metabolic syndrome, T2DM, and obesity, associates with elevated TMAO. TMAO levels are significantly higher in MAFLD patients than controls and correlate positively with disease severity (152). MAFLD patients with obesity show downregulated FXR expression and elevated circulating TMAO and deoxycholic acid (153).

Adipose tissue dysfunction (increased cytokines/chemokines, immune cell infiltration) links to insulin resistance. In mice fed HFD, TMAO supplementation exacerbated glucose intolerance, induced adipose tissue inflammation, and promoted insulin resistance (154). TMAO reduces bile acid pools, inhibiting FXR activity, disrupting bile acid-FXR signaling, and upregulating pathways worsening hepatic steatosis, as confirmed in HFD-fed C57BL/6J mice and human samples (154).

## 7 Alleviating TMAO to treat IBD

An increasing amount of epidemiological evidence suggests that many underlying factors, including high altitude, hypoxia, urbanization, pollution, physical activity, nutrition, and medication, play important roles in the development and progression of IBD (155). Among them, nutrition, as a key controllable factor, has become the main focus of IBD research. This high level of attention stems from its multifaceted potential impact, which not only affects disease risk, but also affects the pathogenesis, course of disease, management strategies, as well as the nutritional and health status of patients (156–158). Diet is recognized as a crucial factor influencing both the onset and progression of IBD, with the ability to directly affect inflammatory processes and immune function, either through direct mechanisms or by modulating the gut microbiota. Consequently, dietary modification emerges as a potentially simple and relatively low-risk strategy to mitigate TMAO-associated IBD.

Fish and seafood, which are typically high in TMAO, have been shown to significantly elevate circulating TMAO levels within 15 minutes of consumption, indicating that dietary TMAO is absorbed directly without the need for microbial conversion (159). Based on this observation, one might infer that increased fish consumption could heighten the risk of IBD. However, contrary to this notion, a growing body of evidence supports the health benefits of fish and seafood consumption, indicating that they may not increase, but rather reduce, the risk of IBD. A systematic review and meta-analysis of observational studies (160) demonstrated an inverse relationship between fish intake and the risk of CD. Moreover, several bioactive compounds found in fish, such as n-3 polyunsaturated fatty acids, taurine, and other active substances, may contribute to the health benefits of seafood. A prospective, population-based cohort study found that consumption of oily fish and fish oil supplements (e.g., EPA and DHA) may serve as protective factors against IBD (161). Additionally, in experimental mouse models of IBD, dietary taurine was shown to protect intestinal epithelial cells, reduce mucosal inflammatory cytokine production, and attenuate colitis (162). Thus, the overall health effects of seafood consumption should be considered in light of nutrient-nutrient interactions. Further research is warranted to gain a better understanding the underlying mechanisms that drive these effects and to clarify the complex relationship between diet, TMAO, and IBD risk (163).

In addition to directly obtaining TMAO from fish and seafood, it is more commonly produced by the gut microbiota through the conversion of foods rich in choline, L-carnitine, and ergothioneine (e.g., red meat, eggs, and mushrooms) (164). This raises the question of whether dietary modification could induce changes in the composition of the microbiome, potentially increasing the abundance of beneficial bacteria and reducing TMAO production. Alternatively, could dietary interventions directly disrupt the TMAO biosynthesis pathway? Long-term adherence to a vegan diet has been associated with a reduced capacity for L-carnitine, a precursor of TMAO, to be converted into TMAO (76). Prebiotics, defined as “selectively fermented components that lead to specific changes in the composition and/or activity of the gastrointestinal microbiota, thereby conferring health benefits to the host” (165, 166), offer a promising strategy. Supplementation with soluble dietary fiber has been shown to increase the abundance of beneficial bacteria while significantly reducing TMA and TMAO metabolism in red meat-fed mice (by 40.6% and 62.6%, respectively). Similarly, long-term adherence to a fiber-rich diet has been linked to reduced TMAO concentrations in obese children, alongside changes in the gut microbiota and improvements in metabolic health (167). Dietary supplementation with resveratrol, a bioactive compound found in wine and grape juice, has also been shown to increase *Lactobacillus* abundance, reduce TMAO levels, and attenuate the atherosclerotic phenotype in ApoE ^(-/-)^ mice fed a high-choline diet (168). Likewise, flavonoids from oolong tea extract and citrus peel promoted *Lactobacillus* growth, reducing the carnitine-induced increase in plasma TMAO levels in mice (169). However, further mechanistic studies and human intervention trials are needed to better elucidate the relationship between diet, microbiota-dependent TMAO production, and its impact on human diseases.

In addition to reducing TMAO intake and synthesis, an anti-inflammatory diet plays an important role in managing IBD. The Mediterranean diet (MD), which emphasizes plant-based foods, olive oil as the primary fat source, limited dairy intake, moderate daily consumption of fish, poultry, and wine, and small amounts of red meat, has been shown to reduce inflammation effectively (170). Recent reviews and meta-analyses have reported that high MD compliance is associated with a significant reduction in chronic disease risk (171). Mechanistically, MD has strong anti-inflammatory effects and enhances the abundance of beneficial gut microbiota (172–174). For example, observational data from healthy adults indicate that the higher the MD adhesion rate, the more SCFAs in feces, and the lower the systemic inflammatory markers (175). Moreover, epidemiological research has demonstrated an inverse association between habitual adherence to the MD and urinary (176) and plasma (177) TMAO levels in southern European populations. Thus, by reducing intake of TMAO precursors and feeding fiber-metabolizing microbes, the MD tends to shift the gut metabolic profile away from TMAO production. Weber et al. investigated various dietary patterns, including a diet low in carbohydrates, with restricted FODMAPs, no gluten, and emphasizing anti-inflammatory properties, and MD, and found that the MD was particularly beneficial for IBD symptom enhancement and mucosal healing (178). Epidemiological and clinical data indicate that MD adherence is beneficial in IBD: higher MD scores are linked to lower IBD incidence (especially CD) and to better disease outcomes, including reduced inflammatory markers, improved clinical scores and even lower mortality. These benefits are attributed to MD’s anti-inflammatory and microbiome-modulating effects. For example, mechanistic studies show MD consumption enriches beneficial gut bacteria (e.g. *Faecalibacterium prausnitzii*, *Roseburia*) and depletes pathobionts (e.g. *Ruminococcus gnavus*), thereby strengthening the gut barrier and immune tolerance (179). Additionally, foods such as soy products, vegetables, fruits, and cereals, along with their bioactive components, have been found to play a significant anti-inflammatory role in the progression of IBD (180–192) (**Table 1**). This compilation of evidence aims to assist IBD patients in selecting foods that may be beneficial during the course of their disease.

**Table 1:** The role of food and its bioactive components in IBD.

Foods	Bioactives	Effects	Mechanisms	References
Soy	Soyasaponins,Phytosterols	Antioxidant,anti-inflammatory and immunomodulatory activity	Inhibited TNF-α,NF-κB,iNOS and COX-2	(180)
Yoghurt	Lactic acid bacteria	Anti-inflammatory and immunomodulatory activity	Increased in the number of the IgA^+^ cells, a decrease in CD8^+^ population	(181)
Fruit	Phenolic acid	Improved intestinal mucosal barrier function, inhibited excessive activation of the immune response, and regulated the balance of the intestinal microbiota	Suppressed p65-NF-κB,NLRP3 and IL-6/p-STAT3 activation	(182)
Cruciferous Vegetables	Glucosinolates	Gut microbiota modulation, anti-inflammatory activity and maintained the intestinal barrier.	Regulation of gut microbiota composition,downregulated inflammatory mediators and inhibited NF-κB	(183)
Mushrooms	HECP,CMP33,LEP	Regulation of inflammatory status, gut microbiota, and immune system and protection of the intestinal epithelial barrier function	Downregulated TNF-α,IL-1β, IL-6,iNOS,COX-2,NO, PGE2,NF-κB,p-p65, NLRP3,Caspase-1	(184)
Purple sweet potato	Purple sweet potato anthocyanins	Maintenance of intestinal homeostasis and protection against bacterial intestinal inflammation	Modulation of gut microbiota	(185)
Quinoa	QPro,QPep	Alleviated colitis symptoms,reduced colonic shortening, inflammatory factor release, and intestinal barrier injury	Suppressed TLR4 levels and inhibited IκB-α and NF-κB phosphorylation	(186)
Olive Oil	HT	Anti-inflammatory	Reduced pro-inflammatory cytokines and chemokines like IL-6,TNF-α and CXCL10/IP-10,inhibited the NF-κB pathway	(187)
Pistachio	γ-tocopherol	Anti-inflammatory,protection of gut barrier integrity and altered gut microbial community	Mitigated elevation of IL-6, inhibited colitis-induced loss of the tight junction protein occluding	(188)
Cocoa and Chocolate	Polyphenols	Antioxidant, anti-inflammatory effects	Activated TLR4/NF-κB/signal transducer and activator of transcription (STAT),modulated intestinal microbiota	(189)
Coffee	Caffeine	Reduced Bacterial translocation into other organs and pro-inflammatory cytokines production	Down-regulation of CHI3L1 expression and its associated bacterial interaction effect	(190)
Green Tea	Green tea polyphenols	Antioxidant, anti-inflammatory	Downregulation of NF-kB, TNF-α, IL-1β and other cytokines	(191)
Ginger	Gingerols	Anti-inflammatory, anti-oxidative	Inhibited NF-κB,STATs, NLRPs,TLRs,MAPKs,mTOR pathways and various pro-inflammatory cytokines	(192)

HECP, polysaccharide of H. erinaceus; CMP33, carboxymethyl polysaccharide; LEP, Lachnum polysaccharide; QPro, quinoa protein; QPep, quinoa peptides; HT, hydroxytyrosol.

## 8 Conclusion

This review summarizes the current evidence on the multifaceted role of TMAO in regulating immunometabolism during IBD. Our analysis reveals that TMAO, generated from dietary precursors via gut microbial metabolism, exacerbates IBD pathogenesis through three interconnected mechanistic pathways. Firstly, there is the ERS pathway mediated by PERK, where TMAO directly binds to and activates PERK, triggering the PERK-eIF2α-ATF4-CHOP axis. This cascade can impair Paneth cell function, disrupt intestinal epithelial integrity, and promote NF-κB driven inflammation by inhibiting PPARγ. The second is the NF-κB inflammatory signaling pathway. TMAO synergizes with LPS to enhance the activation of NF-κB in DCs and macrophages, increase the production of pro-inflammatory cytokines, and impair the function of Tregs. This disrupts the homeostasis of the mucosal barrier and promotes Th17 polarization. The third is the activation of NLRP3 inflammasome. By inhibiting autophagy and inducing mitochondrial ROS, TMAO activates NLRP3 inflammasome, leading to the release and pyroptosis of IL-1β/IL-18 dependent on caspase-1 in colon epithelium, which is a hallmark of IBD severity.

It is crucial that our findings address the apparent paradox in clinical observations. Although TMAO from fish sources can rapidly increase circulating levels, seafood consumption may prevent IBD due to anti-inflammatory nutrients such as n-3 PUFA and taurine, highlighting the context dependent effects of dietary sources. The reduction of TMAO in active IBD patients may reflect the depletion of TMA producing taxa caused by ecological imbalance, rather than ruling out the pathogenic role of TMAO. Targeting the gut microbiota- TMAO axis has positive therapeutic implications. Dietary interventions reduce TMAO by reshaping gut microbiota composition and inhibiting microbial CutC/CytA enzymes. Pharmacological strategies that block PERK, NF-κB, or NLRP3 have reduced TMAO induced inflammation in preclinical models, indicating their translatable potential.

However, current evidence largely relies on animal models. In future research, more human cohort studies can be conducted to correlate TMAO levels with IBD phenotypes through multi-omics analysis. Meanwhile, the bidirectional relationship between TMAO and bile acid metabolism also deserves further exploration, as they jointly regulate FXR signaling. In addition, personalized nutrition methods should consider individual differences in microbial TMAO production capacity and host genetic susceptibility.

In summary, TMAO is both a pathogenic effector and a therapeutic target in IBD. Regulating its production or signaling pathways provides a promising strategy for restoring immune metabolic balance and reducing intestinal inflammation. Future work should prioritize clinical validation of TMAO-lowering interventions and elucidate tissue-specific mechanisms underlying its dual roles in inflammation resolution and exacerbation.
